# Tolerogenic Plasmacytoid Dendritic Cells Control *Paracoccidioides brasiliensis* Infection by Inducting Regulatory T Cells in an IDO-Dependent Manner

**DOI:** 10.1371/journal.ppat.1006115

**Published:** 2016-12-19

**Authors:** Eliseu Frank de Araújo, Daniella Helena Medeiros, Nayane Alves de Lima Galdino, Antônio Condino-Neto, Vera Lúcia Garcia Calich, Flávio Vieira Loures

**Affiliations:** Departamento de Imunologia, Instituto de Ciências Biomédicas, Universidade de São Paulo, São Paulo, SP, Brazil; University of Pittsburgh, UNITED STATES

## Abstract

Plasmacytoid dendritic cells (pDCs), considered critical for immunity against viruses, were recently associated with defense mechanisms against fungal infections. However, the immunomodulatory function of pDCs in pulmonary paracoccidiodomycosis (PCM), an endemic fungal infection of Latin America, has been poorly defined. Here, we investigated the role of pDCs in the pathogenesis of PCM caused by the infection of 129Sv mice with 1 x 10^6^ *P*. *brasiliensis-*yeasts. *In vitro* experiments showed that *P*. *brasiliensis* infection induces the maturation of pDCs and elevated synthesis of TNF-α and IFN-β. The *in vivo* infection caused a significant influx of pDCs to the lungs and increased levels of pulmonary type I IFN. Depletion of pDCs by a specific monoclonal antibody resulted in a less severe infection, reduced tissue pathology and increased survival time of infected mice. An increased influx of macrophages and neutrophils and elevated presence of CD4^+^ and CD8^+^ T lymphocytes expressing IFN-γ and IL-17 in the lungs of pDC-depleted mice were also observed. These findings were concomitant with decreased frequency of Treg cells and reduced levels of immunoregulatory cytokines such as IL-10, TGF-β, IL-27 and IL-35. Importantly, *P*. *brasilienis* infection increased the numbers of pulmonary pDCs expressing indoleamine 2,3-dioxygenase-1 (IDO), an enzyme with immunoregulatory properties, that were reduced following pDC depletion. In agreement, an increased immunogenic activity of infected pDCs was observed when IDO-deficient or IDO-inhibited pDCs were employed in co-cultures with lymphocytes Altogether, our results suggest that in pulmonary PCM pDCs exert a tolerogenic function by an IDO-mediated mechanism that increases Treg activity.

## Author Summary

The fungus *Paracoccidioides brasiliensis* causes paracoccidioidomycosis (PCM), the most relevant deep mycosis in Latin America. The plasmacytoid dendritic cells (pDCs) are important immune cells involved in protection against viral infections, but their role in fungal infections remains unclear. Here, we investigated the role of pDCs in the pathogenesis of pulmonary PCM using a monoclonal antibody to deplete this DC subset. pDCs depletion leads to a less severe PCM associated with increased T cell response mainly mediated by Th1 and Th17 cells. The lung homogenates of depleted mice showed diminished levels of type I IFN and anti-inflammatory cytokines. In addition, a reduced number of regulatory T cells (Treg) paralleled a diminished number pDCs expressing IDO, a potent immunoregulatory enzyme. In agreement, pDCs of IDO^-/-^ mice or IDO-inhibited pDCs stimulated by *P*. *brasiliensis* yeasts expanded elevated numbers of T cells concomitant with a reduced expansion of Treg cells. Taken together, our results demonstrate a tolerogenic activity of pDCs that enhances the severity of a pulmonary mycosis mediated by the concerted action of IDO and Treg cells. These results reveal a new function for pDCs in primary fungal infections and open new perspectives for immunotherapeutic procedures of PCM involving the control of IDO and Treg activity.

## Introduction

*Paracoccidioides brasiliensis*, a thermally dimorphic fungus, is the causative agent of paracoccidioidomycosis (PCM), the most prevalent deep mycosis in Latin America. In humans and murine models of PCM, resistance to disease is associated with the secretion of IFN-γ and other Th1 cytokines, whereas impaired Th1 immunity and the prevalent secretion of Th2 cytokines correlate with systemic and progressive disease [1–3]. The importance of Th17 immunity is not well defined. However, IL-17-expressing cells have been observed in cutaneous and mucosal lesions of PCM patients and have been associated with the organization of granulomas [4]. It was also recently reported that the diverse patterns of T cell responses of *P*. *brasiliensis*-infected individuals lead to different clinical manifestations. The resistance to infection observed in asymptomatic individuals was shown to be mediated by a predominant Th1 response, which is responsible for macrophage activation. The most severe form of the disease, the juvenile form, presents a prevalent Th2/Th9 response and an enhanced antibody response. In the chronic inflammatory response characteristic of the adult form of the disease, a prominent Th17 immunity with important participation of Th1 cells was described [5]. Additional studies with mouse Pattern Recognition Receptors-deficient (PRR-deficient) cells allowed us to demonstrate that dectin-1, mannose receptor (MR), TLR-2, and TLR-4 control lymphocyte proliferation and IL-17 production induced by *P*. *brasiliensis*-stimulated dendritic cells (DCs) [6]. Our *in vivo* studies have also demonstrated that TLR2 deficiency enhanced Th17 immunity, which was associated with diminished expansion of regulatory T cells (Tregs) and increased lung pathology due to unrestrained inflammatory reactions [7]. Furthermore, studies with TLR4, dectin-1 and MyD88 deficient mice led us to demonstrate the essential influence of these receptors and adaptor molecule expression in the control of lung pathology and dissemination of *P*. *brasiliensis* yeasts [8–10].

DCs are bone marrow derived cells that continuously survey their environment for invading microorganisms and are considered professional antigen-presenting cells (APCs) due to their unique ability to activate T cells [11]. Plasmacytoid dendritic cells (pDCs) are a subset of DCs that produce large amounts of type I interferon and are largely involved in the immunity against viral infections [12]. However, pDCs can also exert a tolerogenic function suppressing T cell immunity and expanding regulatory T cells (Treg) in infectious processes and neoplasms [13–16]. Upon viral exposure, pDCs initiate protective antiviral responses by secreting up to 1000-fold more type I IFNs than other cell types [16]. Following TLR stimulation [17, 18] pDCs become potent APCs that secrete high levels of cytokines such as IFN-α and TNF-α and differentiate into mature pDCs upregulating MHC and costimulatory molecules and priming naive T cells to Th1 or Th17 differentiation [17–20].

Beyond the universe of viral infections, an important role of pDCs was demonstrated in the immune response against *Legionella pneumophila*, an intracellular bacterium. These studies showed that pDCs were quickly recruited to the lungs of infected mice, and their depletion led to increased bacterial load in a mechanism independent of type I IFNs production [21]. Furthermore, in some fungal infections an important involvement of pDCs was also described [2, 22–24]. Ramirez-Ortiz showed that pDCs bind to and inhibit the growth of *Aspergillus fumigatus* hyphae and depletion of these cells renders mice hyper-susceptible to experimental aspergillosis [23]. Moreover, our group showed that dectin-2, a C-type lectin receptor (CLR) expressed by human pDCs, acts in cooperation with the FcRγ chain to recognize *A*. *fumigatus* hyphae, activate signaling responses, synthesize TNF-α and IFN-α, and exert antifungal activity. Furthermore, hyphal stimulation of human pDCs triggers a distinct pattern of pDC gene expression and leads to formation of extracellular traps (pETs) [24]. In a previous study, we verified that *in vitro P*. *brasiliensis* infection induced in bone marrow-derived dendritic cells (DCs) of susceptible mice a prevalent inflammatory myeloid phenotype that secreted high levels of IL-12, TNF-α, and IL-β, whereas in resistant mice, a mixed population of myeloid and pDCs secreting inflammatory cytokines and expressing elevated levels of secreted and membrane-bound TGF- β was observed [2].

In the present study, we investigated the contribution of pDCs to host defenses against murine PCM. Depletion of pDCs results in a less severe disease, a high frequency of activated CD4^+^ and CD8^+^ T lymphocytes, concomitant with elevated numbers of macrophages and neutrophils that migrate to the lungs of depleted mice. The analysis of lung homogenates showed diminished levels of type I IFN as well as reduced levels of immunoregulatory cytokines. Furthermore, a reduced number of Foxp3^+^ Treg cells and a decreased presence of pDCs expressing indoleamine 2,3-dioxygenase (IDO), an enzyme with potent immunoregulatory properties, were found in the lungs of pDC-depleted mice. Finally, pDCs of IDO deficient mice and 1MT-treated pDCs stimulated by *P*. *brasiliensis* yeasts were more efficient in the induction and activation of T cells in parallel with reduced expansion of Treg cells. Taken together, our results demonstrate a tolerogenic activity of pDCs associated with increased expansion of Treg cells possibly by an IDO-mediated mechanism.

## Results

### pDC response to *P*. *brasilienis* infection

**Fig 1:**
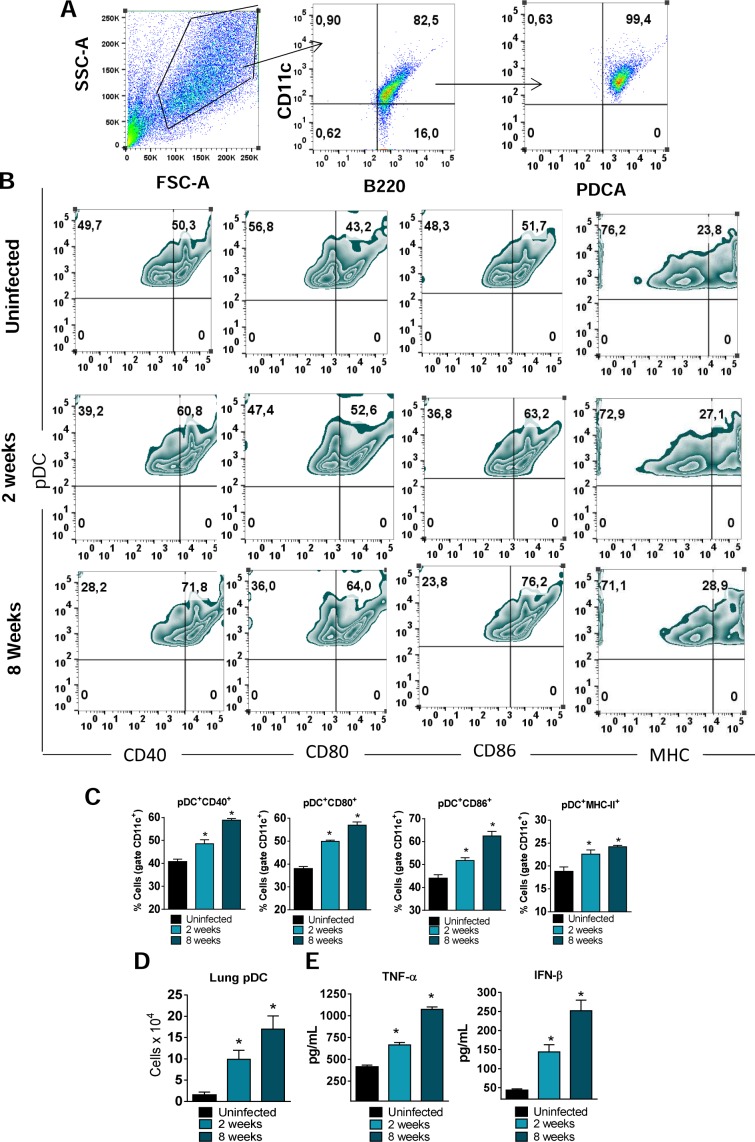
pDC response to *P*. *brasilienis* infection The influx of pDCs to the lungs of *P*. *brasiliensis*-infected mice (1×10^6^ yeasts cells) was determined by flow cytometry at weeks 2 and 8 post-infection. Lungs were removed, leukocytes obtained and the number of pDCs evaluated. The pDCs were characterized as CD11c^+^B220^+^PDCA^+^ cells as indicated in the gate strategy used (A) and the activation measured by the expression of CD40, CD80, C86 and MHC-II molecules on their surface (B-C). The number of pDCs that migrated to the lungs was also determined by flow cytometric analysis (D). The levels of TNF-α and IFN-β were measured by ELISA in pDC supernatants obtained after18 hr of cell culture (E). Data represent the means ± SEM of at least 8 mice and are representative of two independent experiments with equivalent results (*p < 0.05).

Initial experiments were focused on determining the pDCs response following *P*. *brasilienis* infection. pDCs were isolated from the lungs of uninfected and *P*. *brasilienis* infected mice at weeks 2 and 8 post-infection. The maturation of pDCs was assessed by flow cytometric evaluation of activation markers as well a the presence of type I IFNs and TNF-α in the supernatant of cell cultures. A gating strategy to the pDC analysys is represented in the Fig 1A. A mature phenotype of pDCs was induced by *P*. *brasilienis* infection. A higher frequency of cells expressing CD40, CD80, CD86 and MHC-II was observed in pulmonary pDCs isolated from infected mice (Fig 1B and 1C) when compared with cells from uninfected mice. The number of pDCs that migrate to the lungs was also determined. We observed an increased number of pDCs following *P*. *brasilienis* infection (Fig 1D). Regarding cytokines production, pDCs from infected mice produced higher levels of IFN-β and TNF-α (Fig 1E) than pDCs isolated from non-infected mice.

### Depletion of pDCs reduces fungal loads, tissue injury and mortality rates

**Fig 2:**
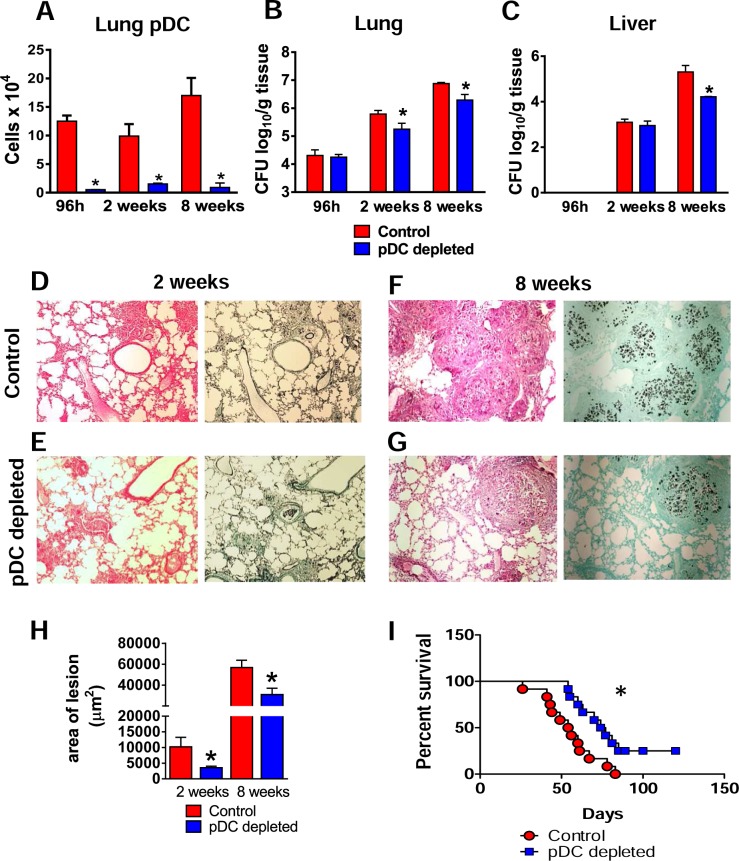
Depletion of pDCs reduces fungal loads, tissue injury and mortality rates. Groups of anti-CD317 (anti-PDCA; clone BX44) or control rat IgG (clone HRPN) treated mice were infected i.t. with 1×10^6^ yeasts cells of *P*.*brasiliensis*. At 96 h, 2 and 8 weeks post-infection lungs were removed, leukocytes obtained and the numbers of pDC analyzed by flow cytometry (A). Colony-forming unit (CFU) counts from lungs (B) and liver (C) were determined 96 h, 2 and 8 weeks after *P*. *brasiliensis* infection. The bars represent means ± standard errors of the mean (SEM) of log_10_ CFU counts obtained from groups of 4–5 mice. (D–G) Photomicrographs of lung lesions of control (D and F) and pDC-depleted mice (E and G) at weeks 2 (D And E) and 8 (F and G) of infection. Lesions were stained with hematoxylin-eosin (left panels) and Grocott (right panels). (See also S1 Fig for liver lesions). (H) Total area of lesions in the lungs at week 2 and 8 of infection. (I) Survival curves of pDC-depleted and control infected mice were determined in a period of 110 days. Data represent the means ± SEM of at least 4 mice/group and are representative of two independent experiments with equivalent results (*p < 0.05).

The severity of fungal infection in pDC-depleted and control groups of *P*. *brasiliensis* infected mice was then assessed at early and late periods of the disease by CFU counts, tissue pathology and mortality rates. The depletion of pDCs by mAb treatment efficiently reduced the afflux of pDCs to the lungs at 96 h, 2 and 8 weeks post-*P*. *brasilienis* infection (Fig 2A). Reduced pulmonary fungal burdens were observed in pDC-depleted mice at weeks 2 and 8 after infection (Fig 2B), although no differences were found at an early period of infection (96 h). Reduced hepatic fungal loads were observed in pDC-depleted mice only at week 8 after infection (Fig 2C). Pulmonary lesions in pDC-sufficient mice (Fig 2D and 2F) replaced large part of normal tissue and were composed of organized granulomas of small sizes although in higher number and containing a higher number of yeasts cells than those found in the pDC-depleted mice (Fig 2E and 2G). As a consequence, smaller lesion areas were observed in the lungs of this latter experimental group (Fig 2H). Reduced liver lesions were also found in pDC-depleted mice (S1 Fig). To assess the influence of pDCs on disease outcome, mortality of infected mice was registered daily. As shown in Fig 2I, at day 82 of infection all control mice were dead and four pDC-depleted mice were still alive.

### pDC-depletion reduces the levels of type I IFNs and anti-inflammatory cytokines but increases inflammatory cytokines and nitric oxide

**Fig 3:**
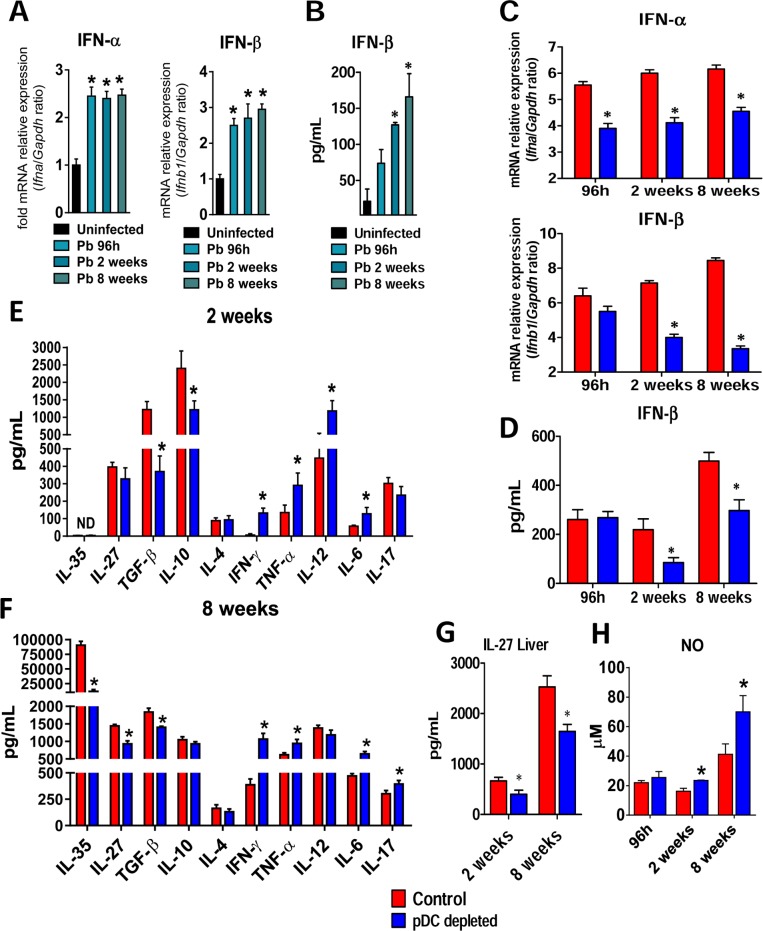
pDC-depletion reduces the mRNA expression of type I IFNs, secreted IFN-β and anti-inflammatory cytokines but increases the production of pro-inflammatory cytokines. (A) Expression of IFN-α and IFN- β mRNA in the lungs of uninfected or *P*. *brasiliensis* infected mice after 96 h, 2 and 8 weeks of infection. (B) Type I IFN quantitation by ELISA in lung homogenates from uninfected and infected mice at the same post-infection periods. (C) mRNA relative expression of IFN-α and IFN- β in the lungs of anti-PDCA or control isotype mAb treated mice. (D) Type I IFN quantitation by ELISA in lung homogenates from pDC-depleted or control mice. (E-F) Cytokines quantitation by ELISA in lung homogenates from pDC-depleted or control mice at weeks 2 and 8 after infection. (G) IL-27 quantitation by ELISA in liver homogenates from pDC-depleted or control mice. (H) Supernatants of lung homogenates were also used to determine the levels of nitrite by the Griess reagent. Bars show mean ± SD from at least four mice per group and are representative of two independent experiments (*p< 0.05).

Because pDCs are major cells involved in the production of type I IFNs [25] the expression of IFN mRNA and proteins was evaluated in the lungs of 129Sv uninfected and *P*. *brasilienis*-infected mice as well as in pDC-depleted and control infected mice. An expressive increase of IFN-α and IFN-β mRNA was observed in the lungs of *P*. *brasilienis* infected mice (Fig 3A) but only IFN-β appeared in detectable levels in the supernatants of lung homogenates (Fig 3B). The levels of IFN-β were consistent with the increased mRNA expression observed at almost all post-infection period studied. A significant reduction of IFN-α and IFN-β mRNA was observed in the lungs of pDC-depleted mice at weeks 2 and 8 of infection (Fig 3C). Again, only IFN-β appeared in detectable levels in the supernatants of lung homogenates of control (IgG) and anti-pDC treated mice, and appeared in reduced levels in pDC-depleted mice (Fig 3D) at weeks 2 and 8 of infection.

By two weeks after infection, lungs of pDC-depleted mice showed diminished levels of regulatory cytokines (TGF-β and IL-10), associated with increased levels of inflammatory cytokines such as IFN-γ, TNF-α, IL-12 and IL-6 (Fig 3E). This behavior suggested that the pDCs were preferably priming regulatory or suppressive T cells and their reduction led to preferential inflammatory responses. By eight weeks after infection, this anti-inflammatory response became more evident: IL-35, IL-27, and TGF-β appeared in reduced levels in contrast to the elevated concentrations of Th1 and Th17 cytokines in the lungs of pDC-depleted mice (Fig 3F). Since IL-27 was previously shown to play important immunoreregulatory functions in the liver, the hepatic synthesis of this cytokine was assessed at weeks 2 and 8 after *P*. *brasiliensis* infection, and reduced levels were found in pDC-depleted mice (Fig 3G). In addition, the hepatic pDCs isolated from infected mice produced higher level of IL-27 than cells of uninfected mice (S2 Fig). Moreover, consistent with the high levels of inflammatory cytokines observed, elevated concentrations of NO were detected in the supernatants of lung homogenates of pDC-depleted mice (Fig 3H).

### Absence of type I IFN signaling increases fungal loads and tissue pathology resulting in increased mortality rates of *P*. *brasiliensis* infected mice

Because pDCs are the major producers of type I IFNs and reduced levels of IFN-β were found in the lung supernatants of pDC-depleted mice, we next examined the susceptibility of IFNα/βR^−/−^ mice, which do not respond to type I IFNs, to *P*. *brasiliensis* infection. To have o more complete view on the effects of IFNs signaling during PCM infection, we also evaluated the course of the disease in the IFNγR^−/−^ and IRF1^-/-^ mice. It is known that IFNs signaling is initiated by two distinct cell-surface receptors, type I IFN receptor (IFNα/βR) and type II IFN receptor (IFNγR). Signaling through IFNαR/STAT1 leads to the formation IFNα-activated factor that mediates the activation of interferon regulatory factor 1 (IRF-1) gene by binding to IFNγ-activated sequence (GAS) in IRF-1 promoter. Likewise, type II IFN signaling through IFNγR/STAT1 also results in STAT1 homodimers binding to GAS and IRF-1 [26–27]. Notably, IRF-1 was the first described member of the family of transcription factors known as Interferon Responsive Factors, which have essential roles in responses against intracellular pathogens, including generation of iNOS and subsequent NO production [28–29].

**Fig 4:**
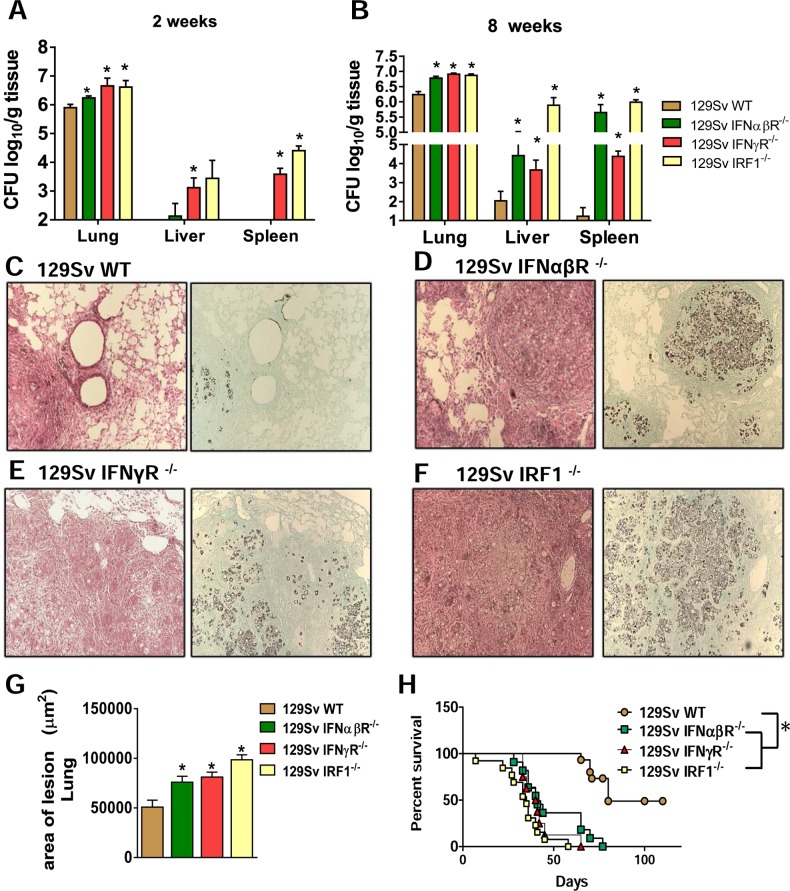
Absence of type I IFN signaling during *P*. *brasiliensis* infection increases mortality rates associated with increased fungal loads and tissue pathology. Colony-forming unit (CFU) counts from organs were determined at 2 (A) and 8 weeks (B) after *P*. *brasiliensis* infection of 129Sv WT, IFNαβR^−/−^, IFNγR^-/-^ and IRF1^-/-^ mice. The bars represent means ± standard errors of the mean (SEM) of log_10_ CFU counts obtained from groups of 4–5 mice. Photomicrographs of lesions of WT mice (C), IFNαβR^−/−^ (D), IFNγR^-/-^ (E) and IRF1^-/-^ (F) mice at week 8 of infection with 1 × 10^6^ *P*. *brasiliensis* yeasts. Staining of lesions was performed with hematoxylin-eosin (left panels) and Grocott (right panels). (G) Total area of pulmonary lesions at week 8 after infection. (H) Survival times of the four studied mouse strains infected i.t. with 1 × 10^6^ *P*. *brasiliensis* yeast cells were determined in a period of 110 days. Data represent the means ± SEM of at least 10 mice/group and are representative of two independent experiments with equivalent results. (*p < 0.05).

The severity of *P*. *brasiliensis* infection was assessed in IFNα/βR^−/−^, IFNγR^−/−^ and IRF-1^-/-^ mice at early and late periods of the disease. Pulmonary, liver and splenic fungal burdens were increased at weeks 2 (Fig 4A) and 8 (Fig 4B) after infection compared to those observed in WT mice. In addition, an increased number of nonorganized lesions containing high numbers of fungal cells and intense tissue destruction were observed in IFNα/βR^−/−^ mice. Pulmonary lesions in IFNα/βR^−/−^ mice replaced large part of normal tissue and were composed of confluent necrotic lesions containing many budding yeasts. The lesions in the lungs of WT mice occupied a small area and were composed of organized granulomas of small sizes (Fig 4C and 4D). The lesions and severity of the disease in the IFNα/βR^−/−^ mice was similar to those found in IFNγR^−/−^ (Fig 4E) and IRF-1^-/-^mice (Fig 4F) as indicated by the area of pulmonary of lesions detected (Fig 4G). Survival of *P*. *brasiliensis*-infected mice was registered daily over a 110-day period (Fig 4H). All deficient strains showed increased mortality rates in comparison with WT mice. The mean survival time of IRF-1^-/-^ mice was 35 days, of IFNα/βR^−/−^ and IFNγR^−/−^ mice was 40 days, while the WT mice presented a mean survival time of 80 days. Altogether these data indicate that type I and type II IFNs are protective to pulmonary PCM, and the less severe disease caused by depletion of pDCs cannot be attributed to the reduced levels of type I IFNs observed during depletion experiments.

### pDC depletion increases the differentiation of type 1 and type 17 CD4^+^ and CD8^+^ T cells

**Fig 5:**
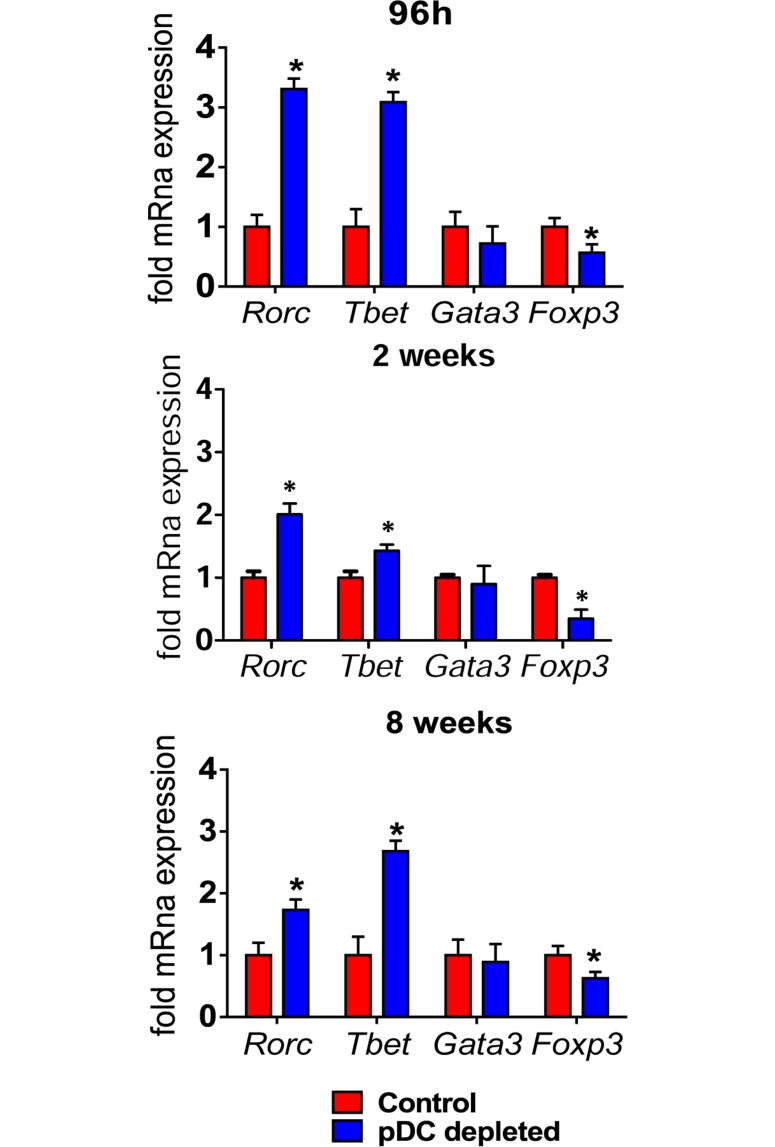
Depletion of pDC induces increased Th1/Th17-related transcription factors and reduced Treg-associated Foxp3. mRNA relative expression of Tbet, Gata3, Rorc and Foxp3 in the lungs of mice treated with anti-mPDCA or control isotype after 96 h, 2 and 8 weeks of infection. Bars show mean ± SD from at least four mice per group and are representative of three independent experiments (*p< 0.05).

**Fig 6:**
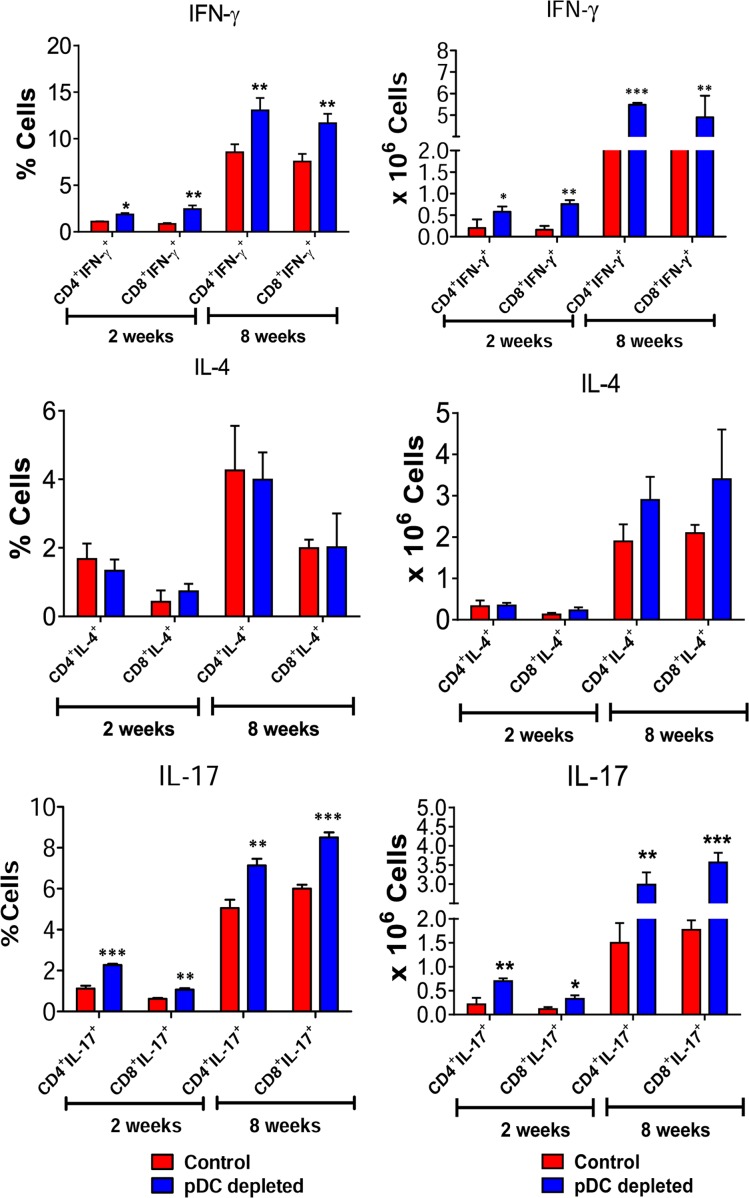
pDC depletion increases the differentiation of type 1 and type 17 CD4^+^ and CD8^+^ T cells. The presence of IL-17^+^, IL-4 and IFN-γ^+^ in CD4^+^ and CD8^+^ T cells in the LILs was assessed by intracellular cytokine staining by flow cytometry at weeks 2 and 8 after infection. Lung cells were stimulated *in vitro* with PMA/ionomycin for 6 hours and subjected to intracellular staining for IL-17 and IFN-γ. The lung infiltrating lymphocytes were gated by FSC/SSC analysis. Lymphocytes were gated for CD4 or CD8 expression and then for IFN-γ, IL-4 and IL-17 expression. Results are expressed in frequency and absolute number of cells and are representative of 3 independent experiments. Data are expressed as means ± SE of the mean. *p < 0.05.

To better clarify the importance of pDCs in the polarization of T cell responses, the expression of genes associated with Treg and T cell subsets was measured by RT PCR in the lungs of pDC-depleted and control mice. As shown in Fig 5, increased mRNA levels of the Th1- and Th17-related transcription factors Tbet and RORγC, and diminished expression of Foxp3 were detected. No significant changes in mRNA levels of GATA3, a Th2-related transcription factor, were detected at all time points analyzed. The polarization of T-cell responses in the inflammatory infiltrates of lungs was also assessed by intracellular staining of IL-17–, IFN-γ– and IL-4-producing cells. Confirming mRNA studies, a higher frequency and number of CD4^+^ and CD8^+^ lymphocytes expressing intracellular IFN-γ and IL-17 were found in the lungs of pDC-depleted mice. No differences were found in lymphocytes expressing intracellular IL-4 between the studied groups (Fig 6).

### pDC depletion determines increased frequencies of pulmonary macrophages, neutrophils and activated T lymphocytes

**Fig 7:**
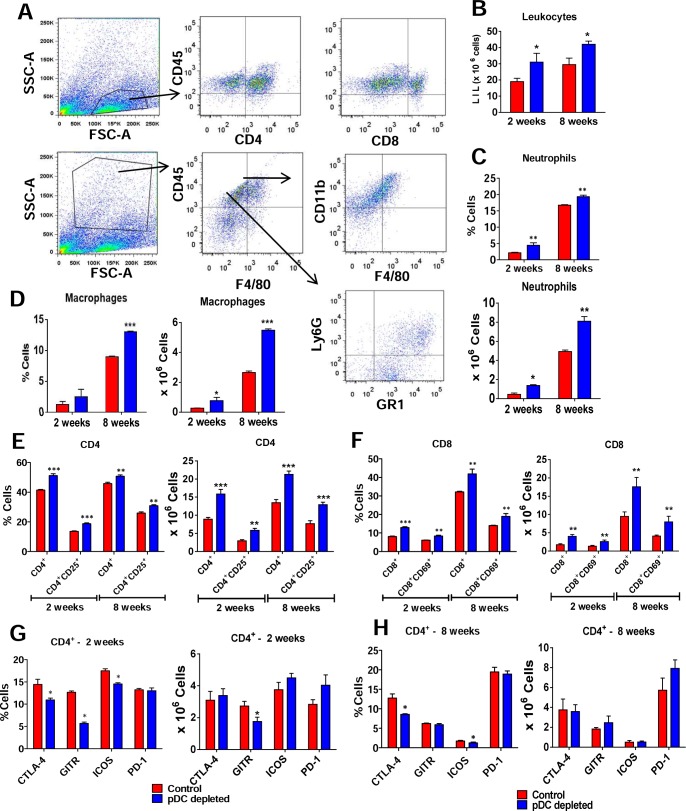
pDC depletion determines increased presence of pulmonary macrophages, neutrophils and activated T lymphocytes. pDC-depleted and control groups were inoculated i.t. with 1 × 10^6^ *P*. *brasiliensis* yeasts and cell phenotypes determined at weeks 2 and 8 after infection. Lungs of both mouse groups (n = 4–5) were excised and digested enzymatically. Cell suspensions were obtained and stained as described in Materials and Methods. The stained cells were analyzed immediately on a FACSCanto II equipment with gating of lymphocytes or granulocytes, as judged from FSC and SSC scatters. Gated CD4^+^ and CD8^+^ cells were also analyzed for the expression of activation and deactivation markers. (A) Representative FACS plots demonstrating the gating strategy for lymphocytes, macrophages and neutrophils. (B) Number of total leukocytes. (C) Frequency and number of neutrophils CD11b^+^F4/80^-^Gr1^+^, and (D) macrophages F4/80^+^CD11b^+^. (E) Total and activated CD4^+^ T and (F) CD8^+^ T cells. Markers of the suppressive activity of T cells (CTLA4, GITR, ICOS, PD-L1) were also measured by flow cytometry at weeks 2 (G) and 8 (H) after infection. One hundred thousand cells were counted and the data expressed as frequency and number of positive cells. Data are expressed as means ± SE of the mean and are representative of three independent experiments. *p < 0.05.

In order to verify whether depletion of pDC affected the influx of effector leucocytes to the lungs, the frequency and activation of macrophages, neutrophils and T cells was assessed by flow cytometry. A gating strategy was represented in the Fig 7A. At weeks 2 and 8 post-infection, an increased number of lung infiltrating leukocytes (Fig 7B) was detected in anti-pDC-treated mice. Compared with controls, a higher frequency and number of neutrophils (Fig 7C), macrophages (Fig 7D), and activated CD4^+^ (Fig 7E) and CD8^+^ T (Fig 7F) cells were observed in the lungs of pDCs depleted mice. In agreement, at week 2 (Fig 7G) post-infection a reduced frequency of infiltrating CD4^+^ T cells expressing deactivation or suppressive molecules (CTLA4, GITR, ICOS) were seen in the lungs of pDC-depleted mice. However, only the number of infiltrating CD4^+^ T cells expressing GITR was reduced in the pDC depleted group. At week 8 post-infection only CTLA4 expressing lymphocytes were observed in decreased frequency (Fig 7H).

### Depletion of pDCs reduces the frequency of pulmonary Treg cells

**Fig 8:**
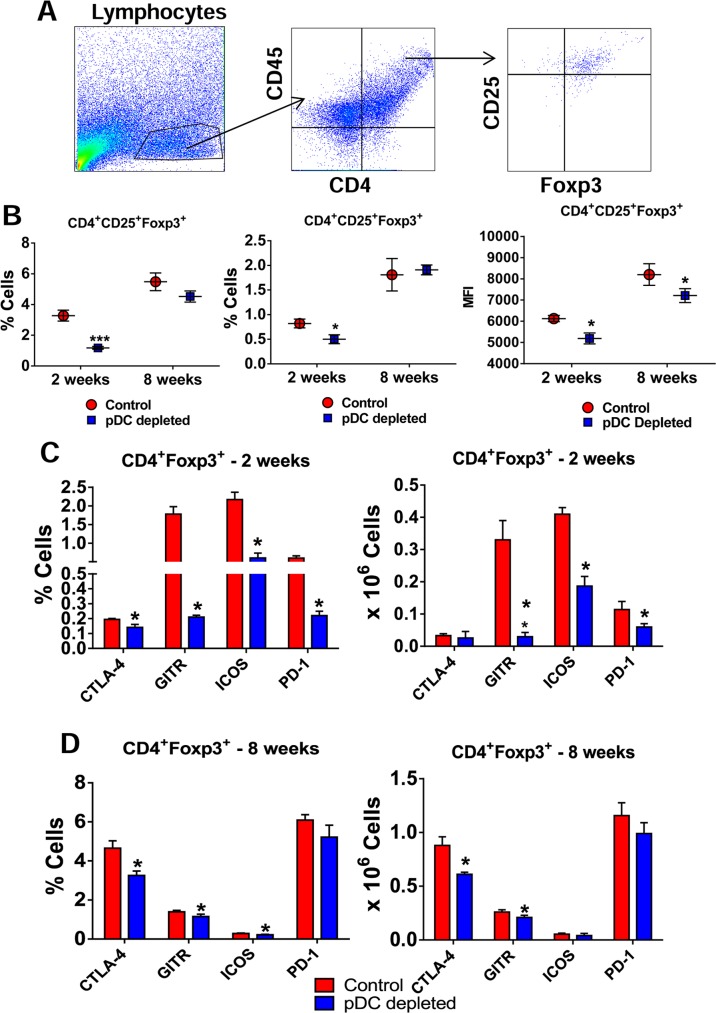
Depletion of pDCs impairs the influx of Treg cells to the lung of *P*. *brasiliensis* infected mice. (A) Representative FACS plots demonstrating the gating strategy for CD4^+^ T and Treg cells. (B) Frequency (left panel), absolute numbers (central panel) and median fluorescence intensity (MFI, right panel) of CD4^+^Foxp3^+^ T cells analyzed by flow cytometry in the lungs of pDC-depleted and control mice after 2 and 8 weeks of infection. Bars reflect mean ± SD of three independent experiments with five mice per group (* *p* < 0.05). (C) Flow cytometric characterization of activation markers of CD4^+^Foxp3^+^ T cells such as CTLA-4, GITR, ICOS, and PD-1 expressed in frequency (left) and number (right) of cells in the lung infiltrating lymphocytes of pDC-depleted and control mice after 2 (C) and 8 weeks (D) of infection. Bars reflect mean ± SD of three independent experiments with five mice per group (* *p* < 0.05).

Since pDCs depletion affected the expansion and migration of T cells, we further characterized the influx of Treg cells to the lungs of treated and control mice. The frequency of CD4^+^ CD25^+^ T cells expressing Foxp3 in the lungs was determined by flow cytometry after 2 and 8 weeks of infection and gated cells in lung homogenates are shown in Fig 8A. pDC-depleted mice showed significantly lower frequency and number of pulmonary CD4^+^CD25^+^Foxp3^+^ Treg cells than IgG-treated control mice (Fig 8B, left and central panels) after 2 weeks of infection. Accordingly, the median fluorescence intensity (MFI) of Foxp3^+^ cells was lower in pDC-depleted mice at both analyzed periods (Fig 8B, right panel). In order to verify whether the reduced expansion of CD4^+^Foxp3^+^ cells was associated with a change in their activation profile, the expression of some activation markers was measured. The Fig 8C and 8D show that at both post-infection periods analyzed, pDC depletion led to decreased frequency and number of CD4^+^Foxp3^+^ Treg cells expressing almost all activation markers studied (CTLA-4, GITR, ICOS).

### IDO contributes to the impaired immune response promoted by pDCs

**Fig 9:**
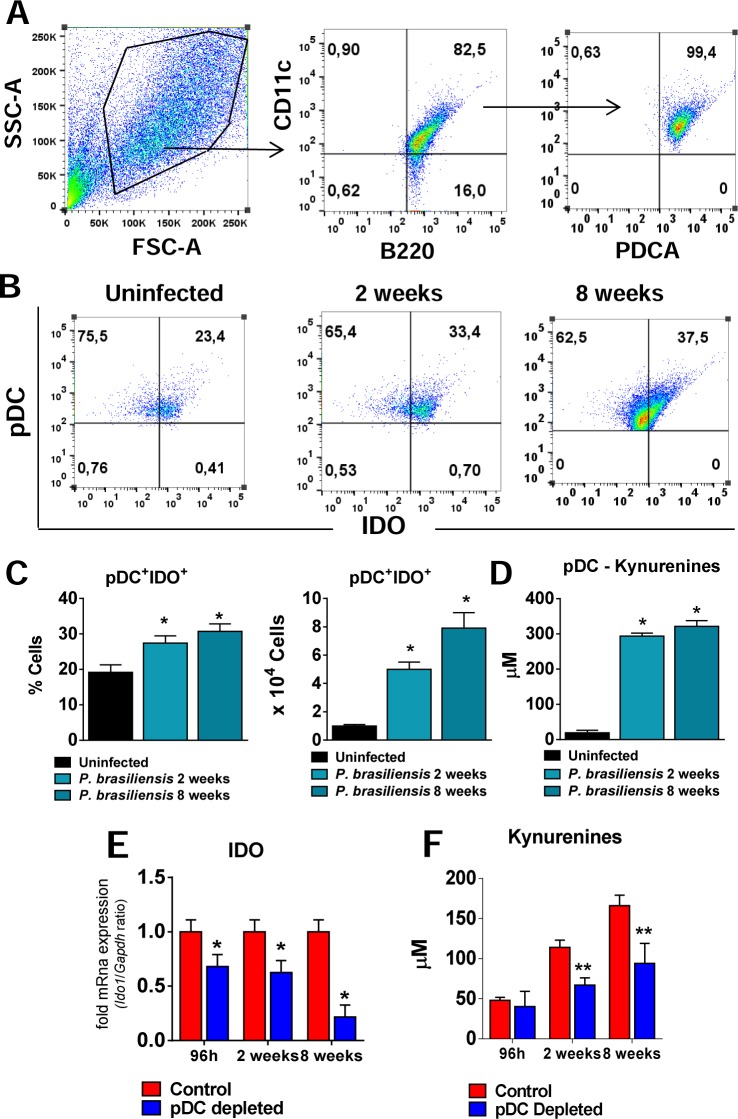
IDO-expressing pDCs and increased levels of kinurenines are observed in the lungs of *P*. *brasiliensis* infected mice. The presence of pDCs expressing intracellular IDO was determined by flow cytometry in the lungs of uninfected and *P*. *brasilienis*-infected mice (1×10^6^ yeasts cells). At 2 and 8 week post infection the lungs were removed, leukocytes obtained and the pDCs isolated by two rounds of positive selection as described in material and methods. The cells were then stained using the Cytofix/Cytoperm kit (BD Biosciences) and specific antibodies anti-IDO. (A) The pDCs were characterized as CD11c^+^B220^+^PDCA^+^ cells as indicated in the gate strategy. (B-C) Plots and bar graphics showing IDO-producing pDCs before and after *P*. *brasiliensis* infection. (D) The pDCs isolated from the lungs were also kept overnight in culture and the kynurenines were measured in the supernatants. Relative expression of IDO mRNA (E) and kynurenines measurements (F) in the lung homogenates of mice treated with anti-mPDCA or control IgG, after 96 h, 2 and 8 weeks of infection. Bars reflect mean ± SD of two independent experiments with five mice per group (* *p* < 0.05).

Our recent study showed that indolemine 2,3 dioxygenase control fungal burdens, and inflammatory reactions in pulmonary PCM [30]. In addition, the production of IDO by pDCs has been linked to the proliferation and activation of resting Foxp3^+^ Treg cells [30, 31]. Our findings demonstrating a reduced frequency of Treg cells in pDC-depleted mice, led us to further investigate the participation of IDO in the tolerance mechanisms promoted by pDCs. First, we evaluated the production of IDO by pDCs isolated from uninfected and infected mice (2 and 8 weeks after *P*. *brasiliensis* infection). A gating strategy to the pDC analysis is represented in the Fig 9A. As can be seen, *P*. *brasileinsis* infection induced an increased frequency and number of pDCs expressing IDO in comparison with uninfected mice (Fig 9B and 9C). Consistent with the IDO production, the levels of kynurenines were also found in increased levels in the supernatants of pDCs isolated from *P*. *brasiliensis* infected mice (Fig 9D).

**Fig 10:**
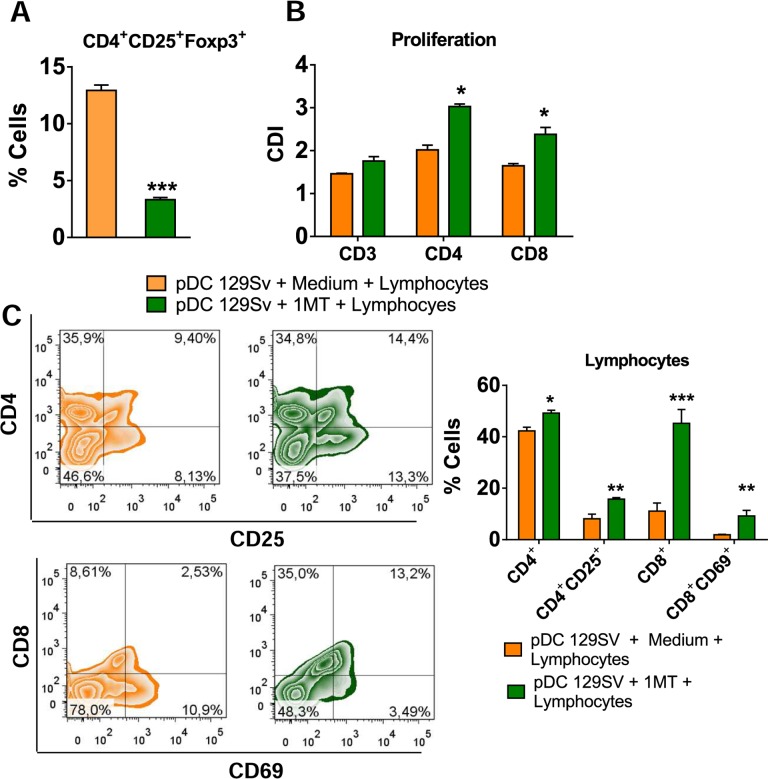
Impaired T cell responses promoted by pDCs is dependent on IDO activity. pDCs were isolated from lungs of uninfected 129Sv mice using magnetics beads anti-mPDCA. The pDCs (5 x 10^4^) were treated or not with 1MT (1mM), matured with *P*. *brasiliensis* yeasts (1:10; Pb:pDC ratio) and then co-cultured for 7 days with splenic CD3^+^lymphocytes (1:10; pDC:lymphocytes ratio) isolated by anti-CD3 magnetic beads from WT mice. (A) Frequency of CD4^+^Foxp3^+^ T cells analyzed by flow cytometry. (B) Splenic lymphocytes from uninfected WT mice were previously labeled with CFSE (5 mM) and co-cultured with *P*. *brasiliensis*-infected pDCs. The co-culture was kept in the presence of RPMI medium containing or not 1MT. After 7 days, the cells were adjusted to 1 × 10^6^, labeled with specific anti-CD4 and CD8 antibodies and analyzed by flow cytometry. (C) After 7 days of co-culture with infected pDCs, lymphocytes were adjusted to 1 × 10^6^, labeled with specific anti-CD4, CD8, CD25, and CD69 antibodies and analyzed by flow cytometry. The lymphocytes were gated by FSC/SSC analysis and gated cells were analyzed for the expression of CD4^+^CD25^+^ (top) and CD8^+^CD69^+^ (bottom). Bars reflect mean ± SD of two independent experiments with eight mice per group (right) (* *p* < 0.05).

Further studies demonstrated a reduced expression of IDO mRNA in the lungs of pDC-depleted mice at all post-infection periods assayed (Fig 9E). In addition, the levels of kynurenines were also found in reduced levels in the lung homogenates of pDC-depleted mice (Fig 9F). Furthermore, to better elucidate the role of IDO in the tolerogenic activity of pDCs, these cells were isolated from non-infected 129Sv mice, treated with 1MT, an IDO inhibitor, infected and matured in the presence of *P*. *brasiliensis* yeasts. These pDCS were then co-cultured with normal splenic lymphocytes from 129Sv mice. Accordingly, the IDO inhibition by 1MT led to a reduced frequency of Treg cells (Fig 10A) associated with enhanced proliferative response of CD3^+^, CD4^+^ and CD8^+^ T cells (Fig 10B), and increased activation of both T cells subsets (Fig 10C). Equivalent results were obtained using the same experimental approach and cells from C57Bl/6 WT and C57BL/6 IDO^-/-^ mice (S3 Fig).

## Discussion

Several studies have demonstrated the important regulatory function of pDCs in immunity against viral infections, autoimmune diseases and in the maintenance of self-tolerance [15, 32, 33]. Although a prominent function of pDCs has been particularly associated with viral infections, recent investigations have expanded this concept to other types of pathogens. Under the universe of microbial infections new data have been added demonstrating an important role of these cells in bacterial [34–36] and fungal infections [2, 23, 24].

In the present study, we investigated the contribution of pDCs to host defenses against pulmonary PCM. Following pulmonary *P*. *brasiensis* infection of 129Sv WT mice, a considerable recruitment of pDCs to the lungs was observed. These recruited pDCs are able to present antigen to T cells and secrete cytokines as evidenced by the hight expression of coestimulatory molecules, MHC-II, TNF-α and IFN-β production. The influx of pDCs to the site of infection was also reported during *A*. *fumigatus* infection [23], but here we were able to demonstrate the mature phenotype of these cells and their ability to secrete cytokines, including type I IFN.

The depletion of pDCs led to a less severe infection, with decreased fungal burdens in the lungs, resulting in increased survival times. This better disease outcome was concomitant with a reduced number of organized lesions containing small numbers of fungal cells and diminished damaged tissue. These findings were opposed to those described previously where pDC-depleted mice were dramatically more susceptible to both pulmonary and systemic infection with *A*. *fumigatus* [23]. Therefore, pDCs can be viewed as protective or detrimental cells during fungal infections, depending on the fungal species that is causing the infection, and further studies with diverse fungal pathogens and morphotypes should be done to better understand the role of pDCs in fungal infections.

pDCs secrete large amounts of type I IFN in response to several viruses and to a large variety of DNA and RNA sequences [37], but their role during fungal infections remains unclear. The role for type I IFNs in invasive aspergillosis was investigated by comparing wild-type mice and IFNα/βR^−/−^ mice. The deficient mice had accelerated mortality after intravenous challenge with *A*. *fumigatus* [23]. In accordance, IFNα/βR^−/−^ mice were susceptible to *Cryptococcus neoformans* and failed to produce protective Th1 cytokines [38], but studies with murine models of candidiasis have implicated type I IFN receptor-deficient mice with decreased survival rates after *Candida albicans* infection [39]. Here we showed that pDC respond to *P*. *brasiliensis* infection producing mRNA to IFN-α and IFN-β, secrete IFN-β at the site of infection but behave as tolerogenic cells that increase disease severity. In agreement, tolerogenic pDCs induced by TGF-β signaling and phosphorylated IDO produce high levels of IDO and TGF-β besides IFN-α/IFN-β in response to the noncanonical NF-;AB pathway of cell activation [32].

During pDC depletion reduced levels of type I IFNs were found in lung homogenates, leading us to hypothesize that the protective mechanism mediated by pDCs depletion could be due to the inhibition of type I IFNs production. To analyze this possibility we have further studied the course of the disease in IFNαβR ^-/-^ mice. A protective effect of type I IFN signaling was detected as demonstrated by the increased fungal loads, tissue pathology and mortality rates developed by deficient mice. It is important to highlight that in depletion experiments only reduced levels of type I IFN were seen whereas total abrogation of type I IFN signaling characterizes the response of type I IFNαβR ^-/-^ mice. Therefore, it is possible that total absence versus partial reduction of type I IFN could explain the apparent discrepancy observed in depletion studies versus experiments using genetically deficient mice. Furthermore, type I IFN signaling is important to nitric oxide (NO) production induced by type 2 nitric oxide synthase (NOS2) [40, 41] and NO has been described as one of the most important mediator involved in the fungicidal mechanisms and immunoprotection against *P*. *brasiliensis* infection [42,43]. Thus, the total absence of type I IFN signaling could have exacerbated more drastic effects in NO production than its partial reduction. It is also relevant to point out that pDC is described as the major source of type I IFNs, but these cytokines can be produced by almost any cell type in the body in response to stimulation of an array of transmembrane and cytosolic receptors [26], but during pDC depletion only one source was withdrawal. Altogether, our data demonstrated that the immunoprotection induced by pDC depletion was not mediated by the inhibition of type I IFN production. Surprisingly, the severity of the disease caused by absence of type I IFN signaling was equivalent to that observed in IFNγR^-/-^ mice. Indeed, IFNγ is a major cytokine associated with PCM resistance due to its macrophage activating activity, induction of NO and inflammatory cytokines such as TNFα resulting in inhibition of *P*. *brasiliensis* replication [1, 3, 44, 45, 46].

Because pDCs express MHC class II molecules as well as costimulatory molecules such as CD40, CD80 and CD86, they can present antigens to CD4^+^ T cells [47, 48]. The pDCs have also been shown to be particularly involved in the differentiation of Th17 cells, an emergent T cell subtype that appears to have an important role in the immunity against fungal infections [49, 50]. In addition, IFN-α produced by pDCs modulated the Th17 differentiation during the early infection of mice by *Bordetella pertussis* [36]. In contrast, in our study pDC depletion increased the differentiation of Th1 and Th17 cells indicating a tolerogenic function of these antigen-presenting cells. This activity, as demonstrated by enhanced expression of mRNA of transcription factors Tbet and RORc for Th1 and Th17 differentiation and production of elevated levels of type-1 and type-17 cytokines by pDC depleted mice, indicates the suppressive activity of this DC subset in the immunity against *P*. *brasiliensis* infection.

The outcome of antigen presentation can be tolerogenic, leading to the differentiation of regulatory or suppressor T cells, T cell anergy and impaired T cell proliferation, depending on the signals developed at the time of antigen recognition [48, 51]. Some reports showed that pDCs resident in human thymus drive natural Treg cell development [52, 53]. Besides, the high expression of some cell markers like ICOS-L provides the maturation of human pDCs and the ability of inducing the differentiation of naive CD4^+^ T cells to Treg IL-10-producing cells [14]. Treg cells can also be induced by PD-L1 signaling [54], and pDCs expressing high levels of this cell component were able to expand an elevated frequency of Treg cells in tolerized recipients. Moreover, splenic pDCs from PD-L1-deficient mice induce greater levels of CD4^+^ T cell proliferation than pDCs from WT mice [31, 55, 56]. In our model, the depletion of pDC affected the expression of Foxp3, the major transcription factor of Treg cells. Accordingly, pDC-depleted mice showed lower frequency Treg cells in comparison with IgG-treated mice, besides a decreased expression of most of cell markers associated with the suppressive activity of Treg cells (CTLA-4, GITR, and ICOS).

In a previous study, we showed that early depletion of Treg cells by anti-CD25 antibodies culminates with a less severe disease in susceptible and resistant mice infected with *P*. *brasiliensis*. Importantly, anti-CD25 treatment led to increased fungicidal mechanisms and increased secretion of Th1/Th2/Th17 cytokines without enhanced tissue pathology [57]. Another study using gain and loss approaches demonstrated the dual role of Tregs that are involved in the control of tissue pathology and in the differentiation of Th17 cells but also in the suppression of protective T cell immunity [58]. The findings here reported are in agreement with our previous studies because we could verify that the impaired Treg response resulted in effective Th1 and Th17 immune response that increased the influx of inflammatory cells to the site of infection without enhanced tissue pathology. This behavior suggests that in pulmonary PCM, pDCs prime and expand regulatory T cells and its deficiency contributes to more efficient Th1 and Th17 immune responses. The measurement of lung cytokines has also demonstrated the importance of pDCs in the production of regulatory cytokines. Indeed, TGF-β, IL-10, IL-35 and IL-27 were found in reduced levels in the lungs of pDC-depleted mice. IL-35 is a recently described Treg cytokine required for maximal regulatory activity of murine and human Treg cells [59]. IL-27 has also broad inhibitory effects on Th1, Th2 and Th17 cells as well as on the expansion of inducible Treg cells [60]. Furthermore, a recent study demonstrated that IL-27 produced by mouse hepatic pDCs has an important contribution to immunoregulation [61]. In agreement, our studies demonstrated a significant augment of IL-27 levels in lungs and liver homogenates after *P*. *brasiliensis* infection as well as a reduction of pulmonary and hepatic IL-27 in pDC-depleted mice. In addition, *ex vivo* experiments confirmed that hepatic pDCs are a source of IL-27. Unfortunately, we did not detect IL-27 in the lung pDCs isolated from uninfected and infected mice. An explanation for this negative finding is the different yield of pDCs recovered from lungs and liver. The *ex vivo* culture of lung pDCs was performed with 1 x 10^5^ cells while 3 x 10^5^ cells were used in the liver pDC cultures. As a whole, the findings here reported confirmed the important tolerogenic role of pDCs in pulmonary PCM. These cells are involved in the expansion of Treg cells, inhibition of Th1 and Th17 immunity but their deficiency do not exert a deleterious effect because the increase inflammatory immunity was accompanied by significant reduction on fungal loads with consequent reduction in fungal-induced pathology.

IDO is a rate-limiting enzyme that converts tryptophan to its metabolic products, collectively known as kynurenines. The expression of IDO by macrophages and DCs subsets allows them to inhibit T cell priming and proliferation associated with enhanced Treg cells differentiation, highlighting the importance of IDO expression in the prevention of hyper inflammatory responses [62]. Besides its enzymatic activity, IDO was also shown to be involved in intracellular signaling events responsible for the amplification and maintenance of a regulatory phenotype in pDCs that promotes tolerance [32, 63, 64]. Our data are in agreement with the axis IDO-Treg in promoting immune tolerance because an increased frequency and number of pDCs expressing IDO during *P*. *brasilensis* infection were found. In addition, a reduced expression of IDO mRNA in the lungs of pDC-depleted mice was detected at all post-infection periods. It was previously described that the IDO activity and sustained production of kynurenines promote the expansion of Treg cells [65]. In accordance, culture supernatants of pDCs isolated from infected mice showed higher levels of kynurenines than those obtained from uninfected mice. In addition, the *in vivo* depletion of pDCs reduces the levels of kynurenines produced indicating a close correlation between IDO expression, kynurenines production and increased expansion of Treg cells.

Our data have also further elucidate the role of IDO in the tolerogenic activity of pDCs. The pDC treatement with 1MT and co-cultured with normal splenic lymphocytes from 129Sv mice led to reduced expansion of Treg cells associated with increased proliferation and activation of T cells. This finding was further confirmed using pDC from IDO^-/-^ mice. In accordance, the frequency of Treg cells was significantly diminished when the pDCs were isolated from IDO-deficient mice than obtained from WT mice. Previous studies highlighted the involvement of a fungal infection in the IDO-dependent promotion of tolerogenic DCs. Candida-hyphae activate the tolerogenic program in some DC subsets and this behavior controls the balance between inflammation and tolerance. This balance is fundamental to the coevolution of host immunity and commensal fungal infections that occur without excessive detrimental effects to both organisms [64, 66]. It is important to note that the main effect of IDO inhibition in the immunity of resistant and susceptible mice previously reported [30] is different from that here reported. In B10.A mice, the IDO activity is mainly mediated by IFN-γ, whereas in the resistant A/J mice IDO is mainly induced by TGF-β signaling [2, 30]. In addition, the early tolerogenic response developed by A/J mice is transitory and later counter balanced by a pro-inflammatory activity and prevalent NLRP3 activation resulting in a late Th1/Th17 immunity tightly regulated by Treg cells [57, 67]. In the 129Sv background, we believe that the tolerogenic role of pDCs appears to be maintained in the course of infection, resulting in more severe infection that can be modulated by pDCs depletion. The disease severity developed by 129Sv mice is more close to that developed by the susceptible mouse strain. However, in contrast to B10.A mice where suppression of T cell response is mediated by excessive pro-inflammatory innate immunity, the deficient T cell immunity here observed was mainly mediated by the sustained expansion of tolerogenic pDCs.

Taken together, our results clearly demonstrated the tolerogenic function of pDCs during pulmonary PCM. These cells are involved in the differentiation of CD4^+^CD25^+^Foxp3^+^ Treg cells possibly by an IDO-mediated mechanism that impairs the development of protective Th1 and Th17 cells. Finally, we believe that these results contribute to a better understanding of immunoregulation during *P*. *brasileinsis* infection, and open perspectives of novel immunotherapeutic procedures based on the control of IDO production and Treg expansion.

## Methods

### Ethics statement

Animal experiments were performed in strict accordance with the Brazilian Federal Law 11,794 establishing procedures for the scientific use of animals, and the State Law establishing the Animal Protection Code of the State of São Paulo. All efforts were made to minimize suffering, and all animal procedures were approved by the Ethics Committee on Animal Experiments of the Institute of Biomedical Sciences of University of São Paulo (Proc.180/11/CEEA).

### Mice

Eight- to 12-week-old male 129Sv WT, 129Sv IFNαβR^-/-^, 129Sv IFNγR^-/-^, 129Sv IRF1^-/-^, C57Bl/6 WT and C57Bl/6 IDO^-/-^ mice were obtained from the specific pathogen free Isogenic Breeding Unit of the Department of Immunology, Institute of Biomedical Sciences, University of São Paulo.

### Fungus and infection

The highly virulent *P*. *brasiliensis* 18 isolate (Pb18) was used throughout this study. Yeast cells were maintained by weekly cultivation in Fava Netto culture medium at 36°C and used on days 5–7 of culture. The viability of fungal suspensions, determined by Janus Green B vital dye (Merck), was always higher than 95%. Mice were anesthetized and submitted to intra-tracheal (i.t.) infection as previously described [68]. Briefly, after intraperitoneal (i.p.) injection of ketamine and xylazine, animals were infected with 1×10^6^ Pb18 yeast cells, contained in 50 μL of PBS, by surgical i.t. inoculation, which allowed dispensing of the fungal cells directly into the lungs.

### pDC depletion

I*n vivo* depletion of pDC with anti-CD317 (PDCA-1) antibodies (BioXcell, USA) was performed as previously described [23]. Briefly, 129Sv WT mice were given i.p. injections of 250 μg of anti-CD317 (clone BX44, BioXcell) or control rat IgG (clone HRPN, BioXcell) diluted in sterile PBS. Antibodies were administered on days -1, O, 1 and every 3 days after infection with *P*. *brasiliensis* yeasts.

### CFU assays, mortality rates, and histological analysis

The numbers of viable microorganisms in cell cultures and infected organs were determined by counting the number of colony-forming units (CFU) as previously described [69]. Mortality studies were done with groups of 10–12 mice. Deaths were registered daily. For histological examinations, the left lung and liver of infected mice was removed and fixed in 10% formalin. Five-micrometer sections were stained by hematoxylin-eosin for an analysis of the lesions and were silver stained (Grocott stain) for fungal evaluation. Morphometrical analysis was performed using a Nikon DXM 1200c camera and Nikon NIS AR 2.30 software. The areas of lesions were measured (in square micrometers) in 10 microscopic fields per slide in 5 mice per group. Results are expressed as the mean ± SEM of total area of lesions for each mouse.

### pDC purification

pDCs were isolated from lungs and liver of 129Sv infected mice after 2 and 8 weeks *of P*. *brasiliensis* infection by two rounds of positive selection, using anti-mPDCA coated magnetic beads (Miltenyi Biotec). The pDCs were counted and used in flow cytometric analysis. Some groups of pDC were cultured overnight (1 x 10^5^/well for lung pDCs; 3 x 10^5^/well for liver pDCs). After 18 hr, the supernatants were removed and stored at -80°C to further measurements of cytokynes and kynurenines.

### Assessment of leukocyte subpopulations and flow cytometric analysis

Lungs and liver from *P*. *brasilienis*-infected 129Sv mice were collected after 2 and 8 weeks of infection. Both organs from uninfected 129Sv mice were also collected. To asses the leukocyte subpopulations after depletion of pDCs, the lungs from 129Sv pDC-depleted and control mice were removed after 96h, 2 and 8 weeks post infection and digested enzymatically for 40 minutes with collagenase (2 mg/mL) in RPMI culture medium (Sigma). Total lung leukocyte numbers were assessed with trypan blue, and viability was always >95%. For cell-surface staining, leukocytes and purified pDCs were washed and suspended at 1 × 10^6^ cells/mL in staining buffer (PBS, 2% fetal calf serum and 0.1% NaN_3_). Fc receptors were blocked by the addition of unlabeled anti-CD16/32 (eBioscience). Leukocytes were then stained in the dark for 20 min at 4°C with the optimal dilution of each monoclonal antibody. To pDC: anti-CD11c, B220, PDCA, CD40, CD80, CD86 and MHC-II; lymphocytes: CD4, CD8, CD25, CD69, CTLA-4, PD-1, ICOS, GITR; macrophages and neutrophils: F4/80, CD11b, Ly6G (eBiosciences or BioLegend). Cells were washed twice with staining buffer, fixed with 2% paraformaldehyde (PFA; Sigma). For intracellular detection of cytokines, leukocytes obtained from lungs were stimulated for 6 hours in complete RPMI medium containing 50 ng/mL phorbol 12-myristate 13-acetate, 500 ng/mL ionomycin (Sigma), and 3 mM monensin (eBioscience). Next, cells were labeled for surface molecules and then treated according to the manufacturer’s protocol for intracellular staining using the Cytofix/Cytoperm kit (BD Biosciences) and specifics antibodies anti-17, IL-4, IFN-γ, FoxP3 and IDO. Cells were washed twice with staining buffer, suspended in 100 μl, and an equal volume of PFA was added to fix the cells. A minimum of 50,000 events was acquired on FACScanto II flow cytometer (BD Biosciences) using the FACSDiva software (BD Biosciences). Lymphocytes, macrophages and neutrophils were gated as judged from forward and side light scatter. For Treg cell characterization, FACS plots or histograms were gated on live CD45^+^CD4^+^CD25^+^ cells and the expression of FoxP3^+^ were determined. The cell surface expression of leukocyte markers as well as intracellular cytokine expression was analyzed using the FlowJo software (Tree Star).

### RNA isolation and cDNA synthesis

Lungs were homogenized in TRIzol reagent using tissue grinders. Phase separation was achieved following addition of 0.2 ml chloroform per ml of TRIzol and centrifugation at 12,000×*g* for 15 min at 4°C. The upper aqueous RNA phase was transferred to a fresh tube and further purified using Ultraclean Tissue & Cells RNA Isolation Kit (MO BIO Laboratories) according to the manufacturer’s protocol. RNA purity and concentration were assessed on a NanoDrop ND-1000 spectrophotometer. An amount of 1 μg total RNA was reverse transcribed in a 20 μl reaction mixture using the High Capacity RNA-to-cDNA kit (Applied Biosystems) following the manufacturer’s instructions.

### Real-time quantitative polymerase chain reaction (RT–PCR)

The cDNA was amplified using TaqMan Universal PCR Master Mix (Applied Biosystems) and pre-developed TaqMan assay primers and probes (IFN-α1, Mm03030145_gH; IFN-β, Mm00439552_s1; Tbet, Mm00450960_m1; GATA3, Mm00484683_m1; RORc, Mm01261022_m1; Foxp3, Mm00475162_m1; IDO, Mm004922590_m1 all from Applied Biosystems). Data were normalized to GAPDH gene expression. PCR assays were performed on an MxP3000P QPCR System and data were developed using the MxPro qPCR software (Stratagene).

### Cytokines and NO detection

Supernatants from cell cultures were separated and stored at −80°C. Lungs and liver from mice uninfected and *P*. *brasiliensis*-infected mice were aseptically removed and individually disrupted in 5 mL of PBS. Supernatants were separated from cell debris by centrifugation at 3,000×*g* for 10 min and stored at -80°C. The levels of IFN-α, IFN-β, IL-4, IL-6, IL-10, IL-12, IL-17, IL-27, IL-35, TNF-α, IFN-γ and TGF-β were measured by capture enzyme-linked immunosorbent assay (ELISA) with antibody pairs purchased from eBioscience or PBL. Nitric oxide production was quantified by the accumulation of nitrite in the supernatants from *in vitro* protocols by a standard Griess reaction [70]. All determinations were performed in duplicate, and results were expressed as micro molar concentration of NO. Plates were read using a spectrophotometric plate reader (VersaMax, Molecular Devices).

### Determination of IDO enzymatic activity

To monitor IDO enzymatic activity from the *ex vivo* pDC culture, kynurenines were measured using a modified spectrophotometric assay [71]. The amount of 50 mL of 30% trichloroacetic acid was added to 100 mL of DCs supernatants, vortexed, and centrifuged at 800 *g* for 5 min. A volume of 75 ml of the supernatant was then added to an equal volume of Ehrlich reagent (100 mg P-dimethylbenzaldehyde, 5 ml glacial acetic acid) in a 96 well microtiter plate. Optical density was measured at 492 nm, using a Multiskan MS (Labsystems) microplate reader. A standard curve of defined L-kynurenine concentrations (0–100 mM) was used to determine unknown kynurenine concentrations.

### Treatment of pDCs with 1MT, lymphocyte purification and co-cultivation

Lung leukocytes were obtained from 129Sv, C57BL/6 WT and C57BL/6 IDO^-/-^ uninfected mice as described above and the pDCs were isolated from cells suspensions by two rounds of positive selection, using anti-mPDCA coated magnetic beads (Miltenyi Biotec). The lymphocytes were obtained from splenic cell suspensions of uninfected 129Sv and C57BL/6 WT mice and the cells isolated by positive selection using anti-CD3 coated magnetic beads (Miltenyi Biotec). The pDCs from 129Sv mice were treated with 1MT (1mM) (Sigma) and then challenged with *P*. *brasiliensis* yeasts at a DC: *P*. *brasiliensis* ratio of 10:1. After 2 h, pDCs were co-cultured with the splenic lymphocytes at a DC:lymphocyte ratio of 1:10 [6] in RPMI media containing or not 1MT. Lymphocyte activation, cell division index (CDI) and the expression of FoxP3 were determined after 7 days of DC-lymphocyte co-culture.

### Lymphocyte proliferation and cell division index (CDI)

Lymphocytes were assayed for proliferation using an *in vitro* fluorescence-based assay. Briefly, 1 × 10^6^ cells from spleens of uninfected C57BL/6 WT mice were stained with 1 μL (5 mM) carboxyfluorescein diacetate succinimidyl ester (CFSE; Molecular Probes) in PBS and 5% fetal calf serum for 15 min at room temperature. CFSE-stained cells were cultured for 7 days with *P*. *brasiliensis*-infected pDCs as described above. Lymphocytes were then stained with anti-CD4 and anti-CD8 antibodies (eBiosciences) and analyzed by flow cytometry as described above. The CDI was calculated as previously described by [72] based on the number of CFSE^+^CD4^+^ or CFSE^+^CD8^+^ T cells found in the stimulated culture/number of CFSE^+^CD4^+^ or CFSE^+^CD8^+^ T cells in the unstimulated culture.

### Statistics

For comparisons of two groups, means ± standard errors were analyzed by the two-tailed unpaired Student *t*-test with the Bonferroni correction applied when making multiple comparisons. For comparisons of greater than two groups, significance was determined using the one- or two-way analysis of variance (ANOVA) with Tukey’s multiple correction. Differences between survivals were compared by log-rank test Calculations were performed using statistical software (GraphPad Prism 5). Statistical significance was defined as *P*<0.05 following corrections.

## Supporting Information

S1 Fig pDC depletion reduces liver injury caused by *P*. *brasiliensis* infection. The severity of fungal infection in pDC-depleted and control groups of *P*. *brasiliensis* infected mice was assessed at weeks 2 (top panels) and 8 of infection (bottom panels) by the histological analysis of liver. Liver lesions of control mice were larger and contained higher number of budding yeast at the eighth week post-infection (C) than at the second week (A). pDC depleted mice showed at both studied periods (B and D) smaller number of lesions containing lower number of yeast cells than control mice (A, C). Photomicrographs of liver lesions of control (A and C) and pDC-depleted mice (B and D) at weeks 2 (A and B) and 8 (C and D) of infection. Lesions were stained with hematoxylin-eosin (left panels) and Grocott (right panels).
(PDF)

S2 Fig *P*. *brasiliensis* infection increases the levels of hepatic IL-27 and the production of IL-27 by isolated liver pDCs. IL-27 quantitation by ELISA in liver homogenates from uninfected and infected mice at weeks 2 and 8 post-infection. pDCs were isolated from uninfected and infected mice, cultivated *ex vivo* (3 x 10^5^ cells/well, 18 h) and supernatants analyzed for the presence of IL-27. Bars show mean ± SD from at least four mice per group and are representative of two independent experiments (*p< 0.05).
(PDF)

S3 Fig Impaired immune response promoted by pDC/Treg interactions is dependent on IDO activity. pDCs were isolated from lung infiltrating leukocytes of uninfected IDO^-/-^ and WT mice using magnetics beads anti-mPDCA, matured with *P*. *brasiliensis* yeasts (1:10; Pb:pDC ratio) and then co-cultured for 7 days with splenic CD3^+^lymphocytes (1:10; pDC:lymphocytes ratio) isolated by anti-CD3 magnetic beads from WT mice. (A) Frequency of CD4^+^Foxp3^+^ T cells analyzed by flow cytometry after 7 days of co-cultivation. (B) Splenic lymphocytes from uninfected WT mice were previously labeled with CFSE (5 mM) and co-cultured with *P*. *brasiliensis* -infected pDCs. After 7 days, the cells were adjusted to 1 × 10^6^, labeled with specific anti-CD4 and CD8 antibodies and analyzed by flow cytometry. (C) After 7 days of co-culture with infected pDCs, lymphocytes were adjusted to 1 × 10^6^, labeled with specific anti-CD4, CD8, CD25, and CD69 antibodies and analyzed by flow cytometry. The lymphocytes were gated by FSC/SSC analysis and gated cells were analyzed for the expression of CD4^+^CD25^+^ (top) CD8^+^CD69^+^ (bottom). Bars reflect mean ± SD of two independent experiments with eight mice per group (right) (* *p* < 0.05).
(PDF)
