# Supplementary material for: Tolerogenic Plasmacytoid Dendritic Cells Control Paracoccidioides brasiliensis Infection by Inducting Regulatory T Cells in an IDO-Dependent Manner
Source: PLoS Pathog. 2016 Dec 19;12(12):e1006115. doi: 10.1371/journal.ppat.1006115 (PMC5215616; doi:10.1371/journal.ppat.1006115)
Supplement: S1 Fig — The severity of fungal infection in pDC-depleted and control groups of P. brasiliensis infected mice was assessed at weeks 2 (top panels) and 8 of infection (bottom panels) by the histological analysis of liver. Liver lesions of control mice were larger and contained higher number of budding yeast at the eighth week post-infection (C) than at the second week (A). pDC depleted mice showed at both studied periods (B and D) smaller number of lesions containing lower number of yeast cells than control mice (A, C). Photomicrographs of liver lesions of control (A and C) and pDC-depleted mice (B and D) at weeks 2 (A and B) and 8 (C and D) of infection. Lesions were stained with hematoxylin-eosin (left panels) and Grocott (right panels). (PDF) [file ppat.1006115.s001.pdf]

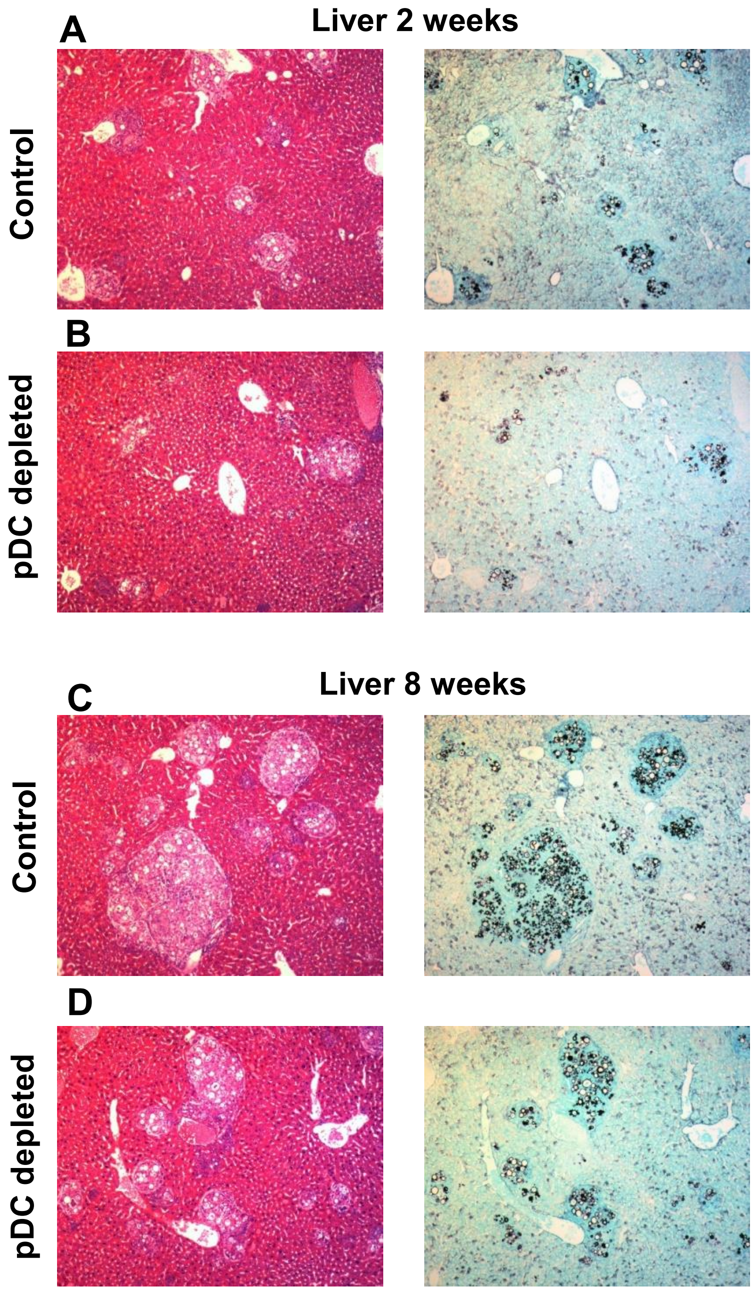

S1 Fig. pDC depletion reduces liver injury caused by *P. brasiliensis* infection. The severity of fungal infection in pDC-depleted and control groups of *P. brasiliensis* infected mice was assessed at weeks 2 (top panels) and 8 of infection (bottom panels) by the histological analysis of liver. Liver lesions of control mice were larger and contained higher number of budding yeast at the eighth week post-infection (C) than at the second week (A). pDC depleted mice showed at both studied periods (B and D) smaller number of lesions containing lower number of yeast cells than control mice (A, C). Photomicrographs of liver lesions of control (A and C) and pDC-depleted mice (B and D) at weeks 2 (A and B) and 8 (C and D) of infection. Lesions were stained with hematoxylin-eosin (left panels) and Grocott (right panels).
