# Supplementary material for: Tolerogenic Plasmacytoid Dendritic Cells Control Paracoccidioides brasiliensis Infection by Inducting Regulatory T Cells in an IDO-Dependent Manner
Source: PLoS Pathog. 2016 Dec 19;12(12):e1006115. doi: 10.1371/journal.ppat.1006115 (PMC5215616; doi:10.1371/journal.ppat.1006115)
Supplement: S2 Fig — IL-27 quantitation by ELISA in liver homogenates from uninfected and infected mice at weeks 2 and 8 post-infection. pDCs were isolated from uninfected and infected mice, cultivated ex vivo (3 x 10^5^ cells/well, 18 h) and supernatants analyzed for the presence of IL-27. Bars show mean ± SD from at least four mice per group and are representative of two independent experiments (*p< 0.05). (PDF) [file ppat.1006115.s002.pdf]

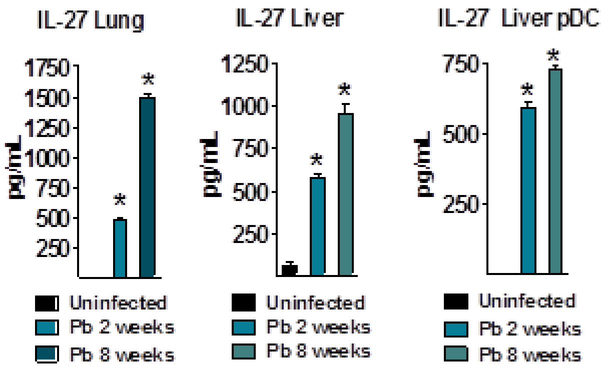

S2 Fig. *P. brasiliensis* infection increases the levels of hepatic IL-27 and the production of IL-27 by isolated liver pDCs. IL-27 quantitation by ELISA in liver homogenates from uninfected and infected mice at weeks 2 and 8 post-infection. pDCs were isolated from uninfected and infected mice, cultivated *ex vivo* (3 x 10^5 cells/well, 18 h) and supernatants analyzed for the presence of IL-27. Bars show mean ± SD from at least four mice per group and are representative of two independent experiments (*p< 0.05).
