# Supplementary material for: Tolerogenic Plasmacytoid Dendritic Cells Control Paracoccidioides brasiliensis Infection by Inducting Regulatory T Cells in an IDO-Dependent Manner
Source: PLoS Pathog. 2016 Dec 19;12(12):e1006115. doi: 10.1371/journal.ppat.1006115 (PMC5215616; doi:10.1371/journal.ppat.1006115)
Supplement: S3 Fig — pDCs were isolated from lung infiltrating leukocytes of uninfected IDO-/- and WT mice using magnetics beads anti-mPDCA, matured with P. brasiliensis yeasts (1:10; Pb:pDC ratio) and then co-cultured for 7 days with splenic CD3+lymphocytes (1:10; pDC:lymphocytes ratio) isolated by anti-CD3 magnetic beads from WT mice. (A) Frequency of CD4+Foxp3+ T cells analyzed by flow cytometry after 7 days of co-cultivation. (B) Splenic lymphocytes from uninfected WT mice were previously labeled with CFSE (5 mM) and co-cultured with P. brasiliensis -infected pDCs. After 7 days, the cells were adjusted to 1 × 10^6^, labeled with specific anti-CD4 and CD8 antibodies and analyzed by flow cytometry. (C) After 7 days of co-culture with infected pDCs, lymphocytes were adjusted to 1 × 10^6^, labeled with specific anti-CD4, CD8, CD25, and CD69 antibodies and analyzed by flow cytometry. The lymphocytes were gated by FSC/SSC analysis and gated cells were analyzed for the expression of CD4+CD25+ (top) CD8+CD69+ (bottom). Bars reflect mean ± SD of two independent experiments with eight mice per group (right) (* p < 0.05). (PDF) [file ppat.1006115.s003.pdf]

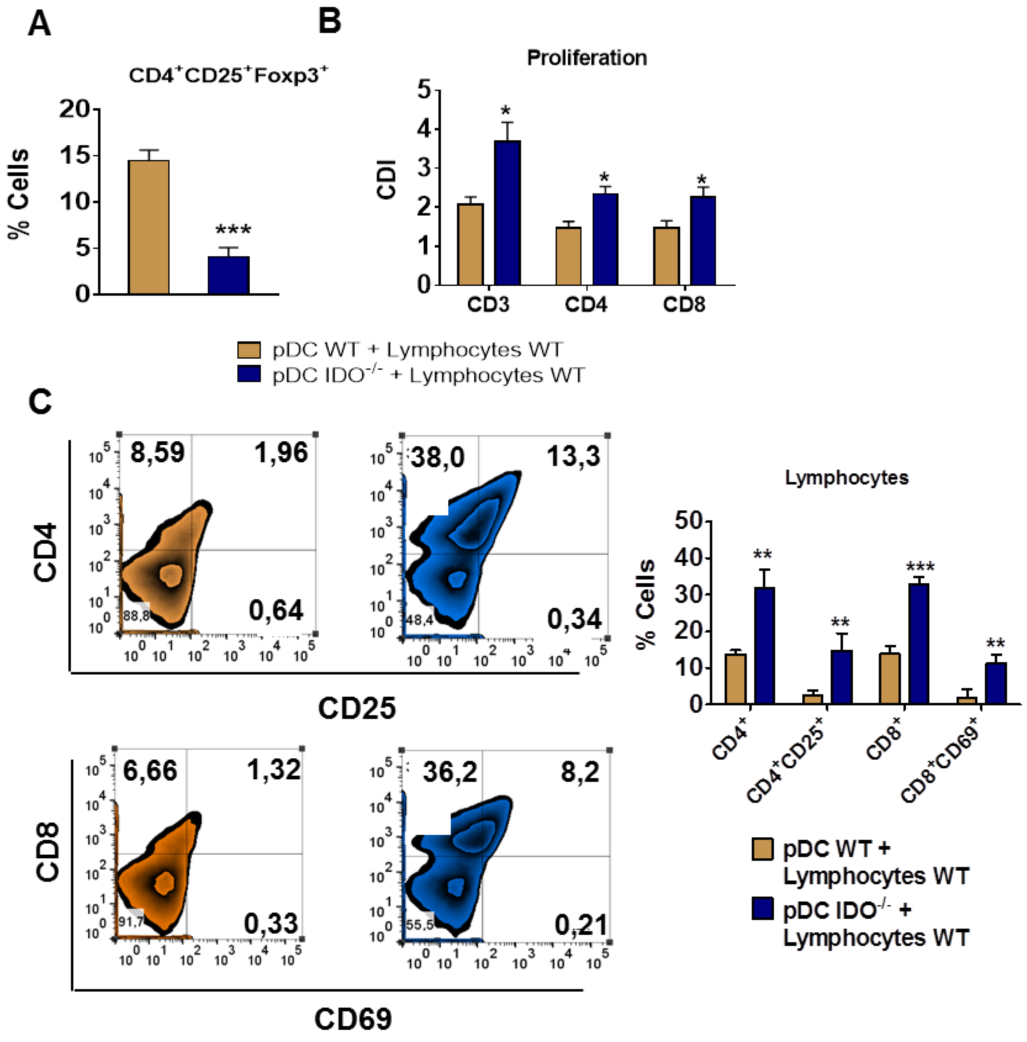

S3 Fig. Impaired immune response promoted by pDC/Treg interactions is dependent on IDO activity. pDCs were isolated from lung infiltrating leukocytes of uninfected IDO$^{-/-}$ and WT mice using magnetics beads anti-mPDCA, matured with *P. brasiliensis* yeasts (1:10; Pb:pDC ratio) and then co-cultured for 7 days with splenic CD3^{+}lymphocytes (1:10; pDC:lymphocytes ratio) isolated by anti-CD3 magnetic beads from WT mice. (A) Frequency of CD4^{+}Foxp3^{+} T cells analyzed by flow cytometry after 7 days of co-cultivation. (B) Splenic lymphocytes from uninfected WT mice were previously labeled with CFSE (5 mM) and co-cultured with *P. brasiliensis* -infected pDCs. After 7 days, the cells were adjusted to 1×10^6, labeled with specific anti-CD4 and CD8 antibodies and analyzed by flow cytometry. (C) After 7 days of co-culture with infected pDCs, lymphocytes were adjusted to 1×10^6, labeled with specific anti-CD4, CD8, CD25, and CD69 antibodies and analyzed by flow cytometry. The lymphocytes were gated by FSC/SSC analysis and gated cells were analyzed for the expression of CD4^{+}CD25^{+} (top)

$CD8^+CD69^+$ (botton). Bars reflect mean ± SD of two independent experiments with eight mice per group (right) (* $p < 0.05$).
